# Effect of plasma exchange on in-hospital mortality in patients with pulmonary hemorrhage secondary to antineutrophil cytoplasmic antibody-associated vasculitis: A propensity-matched analysis using a nationwide administrative database

**DOI:** 10.1371/journal.pone.0196009

**Published:** 2018-04-23

**Authors:** Eishi Uechi, Masato Okada, Kiyohide Fushimi

**Affiliations:** 1 Department of Health Policy and Informatics, Tokyo Medical and Dental University Graduate School, Yushima, Bunkyo-ku, Tokyo, Japan; 2 Division of Rheumatology, Tomishiro Central Hospital, Ueta, Tomigusuku-shi, Okinawa, Japan; 3 Immuno-Rheumatology Center, St. Luke’s International Hospital, St. Luke’s International University, Akashi-cho Chuo-Ku, Tokyo, Japan; Postgraduate Medical Institute, INDIA

## Abstract

**Background:**

Secondary pulmonary hemorrhage increases the risk of mortality in patients with antineutrophil cytoplasmic antibody-associated vasculitis (AAV); plasma exchange therapy may improve outcomes in these patients. We conducted a retrospective cohort study to investigate the effect of plasma exchange therapy on short-term prognoses in patients with pulmonary hemorrhage secondary to AAV.

**Methods:**

This study utilized the Diagnosis Procedure Combination database, which is a nationwide inpatient database in Japan. We checked the abstract data and medical actions and identified the patients with pulmonary hemorrhage secondary to AAV who required proactive treatment between 2009 and 2014. To compare the in-hospital mortality, we performed propensity score matching between the plasma exchange and non-plasma exchange groups at a ratio of 1:1.

**Results:**

Of the 52,932 patients with AAV, 940 developed pulmonary hemorrhage as a complication. A total of 249 patients from 194 hospitals were eligible for the study. Propensity score matching at a ratio of 1:1 was performed, and 59 pairs were formed (plasma exchange group, n = 59; non-plasma exchange group, n = 59). A statistically significant difference was found in the all-cause in-hospital mortality between the plasma exchange and non-plasma exchange groups (35.6% vs. 54.2%; p = 0041; risk difference, −18.6; 95% confidence interval (CI), −35.4% to −0.67%).

**Conclusion:**

Thus, plasma exchange therapy was associated with improved in-hospital mortality in patients with pulmonary hemorrhage secondary to AAV.

## Introduction

Antineutrophil cytoplasmic antibody-associated vasculitis (AAV) is a disease that affects various organ systems via the generalized vasculitis of small blood vessels [1,2]. Patients with AAV and secondary pulmonary hemorrhage (PH) have an increased mortality risk, which is 8.65-fold greater than that of patients with AAV without secondary PH. Hence, PH presents a great risk for early death in these patients [3]. The European League Against Rheumatism, European Renal Association, and European Dialysis and Transplant Association stated that the addition of plasma exchange (PE) therapy should be considered in patients with severe PH [4]. However, this treatment recommendation is only based on a case series study from a few institutions [5]. Various studies that have comparatively assessed PE therapy in PH secondary to AAV did not confirm a reduced mortality rate [6–10].

AAV is a rare disease, with a prevalence of approximately 20 per 100,000 people, as reported in a European study [11]. The incidence of PH secondary to AAV is even lower, occurring in 6.4–36% of patients with AAV [6, 7, 9, 10, 12]. Among these patients, 31–88% require supplementation with oxygen and proactive treatments [6, 7, 13, 14]. As such, it is difficult to conduct a multicenter comparative study to assess the prognosis and acute-phase life expectancy of patients with PH secondary to AAV who require proactive treatment. Therefore, we assessed the effect of adding PE therapy to the treatment for PH secondary to AAV on the acute-phase prognosis, using a Japanese nationwide inpatient information database.

## Materials and methods

### Data source: Diagnosis Procedure Combination database

This study was performed using the Diagnosis Procedure Combination (DPC) database. DPC data comprise treatment costs and patient information at discharge [15] from all university hospitals and many acute-care community hospitals. The participating hospitals submitted all the data on the discharged patients to the DPC study group. The number of participating hospitals was 1,329, representing 50% of all acute care hospitals in Japan [16, 17]. DPC data contain the age, sex, type of hospital (academic or non-academic), ICD-10 codes of the diagnoses, comorbidities at admission, and complications after admission. Furthermore, these data contain records of the drugs required and blood preparations used daily for the in-hospital treatments, devices used, number of in-hospital days, and outcomes at discharge [18, 19]. The data for each patient are confirmed by the respective attending physician at the time of discharge. Moreover, the diagnosis category must be documented in the medical charts [19]. Only in-hospital data were used in this study, which included data from the day of admission to the day of discharge. We were unable to follow up with the patients after their discharge from the hospital. In many hospitals in Japan, recovery and rehabilitation are performed in the same hospital following acute treatment. The entire treatment period is considered to be a single hospitalization; therefore, patients’ in-hospital stays tend to be long-term [20, 21].

This study was approved by the institutional review board of Tokyo Medical and Dental University. As the patients were anonymized in the data registry, the requirement for informed consent was waived.

### Patient selection and data

From the 2009 to 2014 DPC databases, we extracted the in-hospital data of patients with the following AAV diseases: AAV (ICD-10 code: M318), antineutrophil cytoplasmic antibody-related nephritis (N017), microscopic polyangiitis (M300), eosinophilic granulomatosis with polyangiitis (M301), granulomatosis with polyangiitis (M313), and PH (R048).

The study population was limited to patients requiring acute treatment for PH secondary to AAV. The exclusion criteria were as follows: (1) non-use of oxygen supplementation; (2) patients who did not receive steroids during hospitalization; (3) patients with a comorbidity that triggers respiratory tract hemorrhage or a disease that required oxygen therapy (for example, patients with acute respiratory infections, tuberculosis, malignant respiratory tumors, or Goodpasture syndrome [5–7, 9, 10, 13, 22]); (4) patients with unknown outcomes at discharge because of a transfer to another acute hospital; and (5) patients whose treatment duration could not be ensured or who were discharged within 5 days.

The primary endpoint was in-hospital death due to all causal factors. The secondary endpoints were 30 and 60 days in-hospital mortality, incidence of infections during the in-hospital stay, and length of the hospital stay.

Baseline data (obtained from the DPC) included age, sex, type of hospital (university hospital or educational institution approved by the Japan College of Rheumatology), emergency hospitalization, and comorbidities at admission. The five-factor score (FFS) [23] of the predictive indicators of prognosis, calculated based on the diagnoses, was prepared using the ICD-10 codes [15]. The confirmed comorbidities included neurological disorders, diabetes, and malignancies. Furthermore, we extracted the following data on clinical management after admission: use of the intensive care unit (ICU) for in-hospital interventions, use of mechanical ventilators, hemodialysis, corticosteroid treatment, steroid pulse therapy [24], cyclophosphamide treatment, rituximab treatment, and use of PE therapy.

### Statistical analysis

Several retrospective studies have demonstrated that PE therapy tends to be selected for very sick patients [6, 13]. Since this is a non-randomized retrospective study, comparing unadjusted baseline data will likely lead to “confounding by indication” and underestimation of the effect of PE therapy. Thus, an analysis based on propensity score matching was performed to adjust for the confounding factors [25]. To estimate the propensity score, a logistic regression model was used with the factors affecting the decision to perform PE as variables. The covariates used were sex, age, educational institutions certified by the Japan College of Rheumatology, university hospitals, emergency hospitalization, type of AAV diagnosis, FFS [23], complications at admission (maintenance dialysis, interstitial pneumonia, diabetes, neurological disorders, malignancies, and chronic pulmonary diseases), use of mechanical ventilation, blood transfusions, use of the ICU, pulse steroid therapy, cyclophosphamide, and rituximab. A 1:1 matching between the PE and non-PE groups was performed based on the propensity score, using the closest value within the caliper (0.05). The model goodness-of-fit was evaluated by calculating the C-statistic [26]. The chi-square test was used to evaluate group differences in categorical variables, and the Student’s t-test or Mann-Whitney U test was used to evaluate group differences in continuous variables. Multiple logistic regression analysis was performed to explore the predictors of in-hospital mortality. Stepwise analysis was not performed, and variables with a p-value <0.1 in the univariate analysis comparing survivors and non-survivors were selected. A p-value of 0.05 was set as the level of statistical significance. The software used for all statistical analyses was JMP version 12.0 (SAS Institute Inc., Cary, NC, USA).

## Results

### Study patients

**Fig 1:**
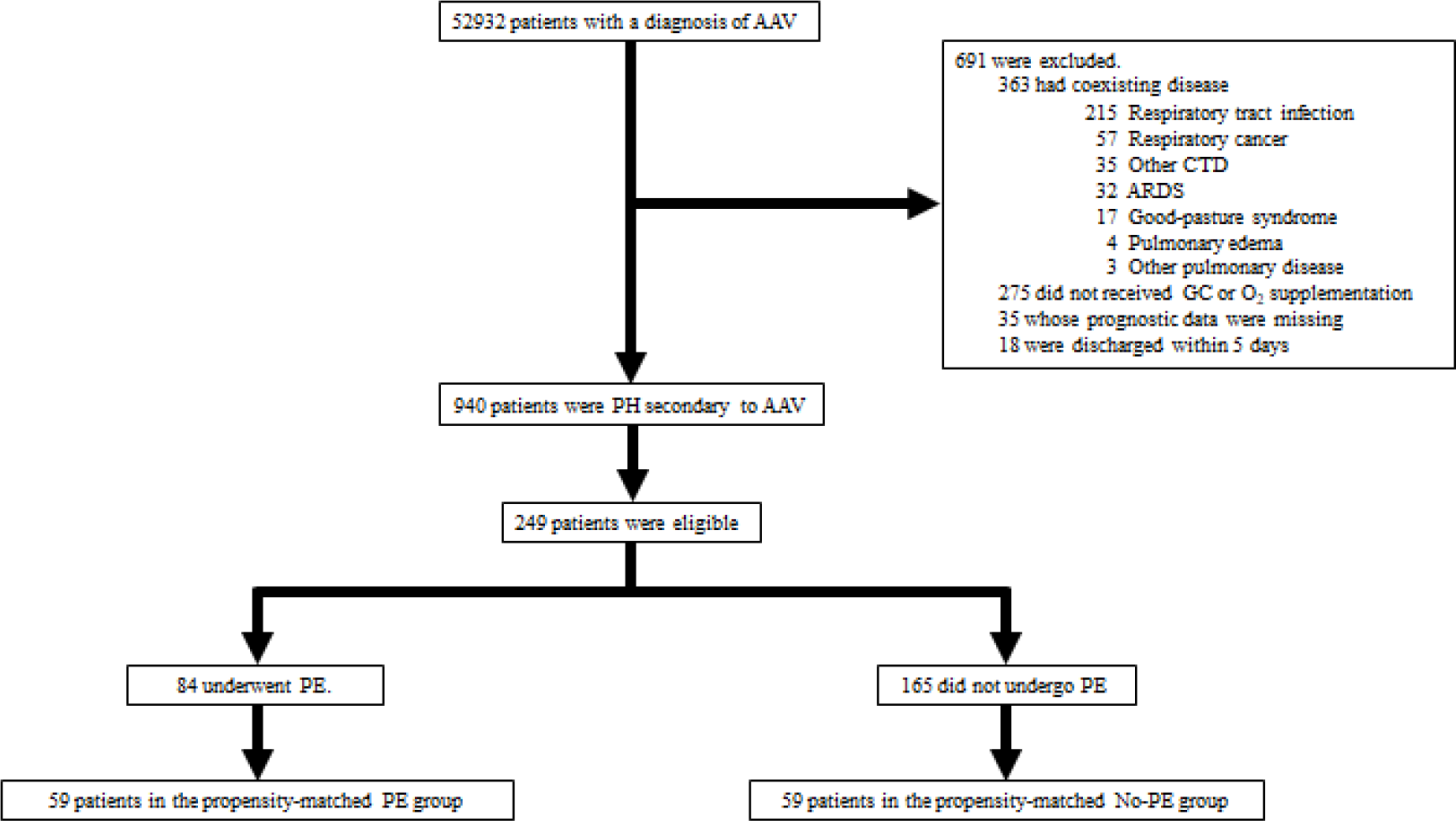
Patient selection flowchart. The patient selection procedure is depicted. AAV, antineutrophil cytoplasmic antibody-associated vasculitis; PH, pulmonary hemorrhage; CTD, connective tissue disease; GC, glucocorticoid; O_2_, oxygen; PE, plasma exchange.

Fig 1 illustrates the flowchart of the patient selection procedure. The number of patients with AAV in the study period was 52,932, of which 940 patients experienced PH. The study was restricted to patients on oxygen and steroid therapy and those using mechanical ventilation. Therefore, the final number was 249 patients from 194 hospitals. Fifty-nine pairs of patients were formed based on a 1:1 propensity matching. The C-statistic was 0.80, indicating a strong goodness-of-fit.

### Baseline characteristics and clinical outcomes

Table 1 presents the baseline characteristics of the patients, interventions, and outcomes prior to matching. The mean age of the patients was 70.4 years, with male patients constituting 55.8% of the patients (n = 139). The categories of AAV diagnoses were microscopic polyangiitis (n = 133, 54.2%), unclassified (n = 93, 37.8%), granulomatosis with polyangiitis (n = 20, 8.0%), and eosinophilic granulomatosis with polyangiitis (n = 1, 0.4%).

**Table 1:** Clinical characteristics, management, and outcomes.

Male patients, n (%)	139 (55.8)
Age, median (IQR) (years)	72 (65.5–78)
Hospital type, n (%)	
Academic	69 (28.3)
JCR-certified educational facilities	164 (67.2)
Diagnosis, n (%)	
MPA	135 (54.2)
GPA	20 (8.0)
EGPA	1 (0.4)
Unspecified AAV	93 (37.8)
Organ damage, n (%)	
Nephritis	176 (70.3)
Maintenance dialysis	55 (22.0)
ENT	12 (4.8)
Interstitial pneumonia	62 (24.9)
Five-factor score	
0	12 (4.8)
1	57 (22.9)
≧2	180 (72.3)
Coexisting disease, n (%)	
Chronic pulmonary disease	20 (8.0)
DM	74 (29.7)
Neurological disorder	18 (7.2)
Malignancy	22 (8.3)
Charlson comorbidity index, n (%)	
0–1	91 (36.5)
2–3	120 (48.2)
≧4	38 (15.3)
Management	
Blood transfusion, n (%)	99 (39.8)
Oxygen supplementation	249 (100)
Mechanical ventilation, n (%)	107 (43.0)
Invasive, n (%)	103 (41.4)
Noninvasive, n (%)	4 (1.6)
Duration of invasive mechanical ventilation, median (IQR) days	9 (6–21)
ICU admission, n (%)	62 (24.9)
Length of ICU stay, median (IQR) days	9 (4–14)
Temporary dialysis, n (%)	13 (5.2)
Initiation of maintenance dialysis, n (%)	31 (12.5)
Corticosteroids, n (%)	
Oral corticosteroid	24 (9.6)
IV corticosteroid	225 (90.4)
Pulse steroid	215 (86.3)
Remission induction treatment, n (%)	
Cyclophosphamide	108 (43.3)
Rituximab	7 (2.8)
Plasma exchange, n (%)	84 (33.7)
Plasma exchange sessions, median (IQR) no.	3 (3–6)
Outcome	
Overall in-hospital mortality, n (%)	92 (36.9)
30-day in-hospital mortality, n (%)	50 (20.1)
60-day in-hospital mortality, n (%)	76 (30.5)
Date of death, median (IQR) days	27.5 (14.3–49.5)
Length of hospital stay, median (IQR) days	40 (23–64)

JCR, Japan College of Rheumatology; MPA, microscopic polyangiitis; GPA, granulomatosis with polyangiitis; EGPA, eosinophilic granulomatosis with polyangiitis; AAV, antineutrophil cytoplasmic antibody-associated vasculitis; ENT, ear, nose, and throat; DM, diabetes mellitus; IV, intravenous; IQR, interquartile range; ICU, intensive care unit

Fifty-five (22%) patients had end-stage renal disease or were on maintenance dialysis upon admission. The number of patients requiring treatment in the ICU was 62 (24.9%) and those requiring mechanical ventilation were 107 (43.0%). A total of 84 (33.7%) patients were treated with PE therapy. The total number of in-hospital deaths was 92 (36.9%). The number of patients who required dialysis after admission was 99 (39.4%). In addition, 31 patients (12.5%) were on maintenance dialysis, and 13 patients (5.2%) stopped dialysis in the hospital.

### PE group vs. non-PE group

Table 2 presents the baseline characteristics of both groups before and after the propensity score matching. In the pre-matched cohort, the PE group had a higher percentage of patients who were on mechanical ventilation (61.9% vs. 33.3%), required treatment in the ICU (41.7% vs. 16.4%), required blood transfusions (53.6% vs. 32.7%), had a FFS ≥2 (82.1% vs. 67.3%), were on pulse steroid therapy (97.6% vs. 87.2%), and were on cyclophosphamide treatment (54.8% vs. 37.6%) compared to those in the non-PE group. On the other hand, the PE group had a lower percentage of patients with GPA (1.2% vs. 11.5%) and those with interstitial pneumonia (16.7% vs. 29.1%). After the propensity score matching, the groups were balanced with respect to all of these baseline characteristics.

**Table 2:** Baseline patient characteristics in pre-matched and propensity-matched groups.

	Pre-matched cohort			Matched cohort		
	PE (n = 84)	Non-PE (n = 165)	p	PE (n = 59)	Non-PE (n = 59)	p
Male sex, n (%)	4 (57.1)	91 (55.2)	0.76	36 (61.0)	35 (59.3)	0.85
Age, median (IQR) years	73 (67–77)	72 (65–78)	0.4	73 (65–76)	74 (67–79)	0.2
Hospital type, n (%)						
JCR-certified educational facilities	56 (67.5)	108 (67.1)	0.95	39 (66.1)	38 (65.3)	0.85
Academic hospital	25 (30.1)	44 (27.3)	0.65	18 (30.5)	15 (25.4)	0.54
Diagnosis, n (%)						
MPA	51 (60.7)	84 (50.9)	0.14	34 (57.6)	38 (64.4)	0.45
GPA	1 (1.19)	19 (11.5)	0.005	1 (1.7)	1 (1.7)	1
Unspecified AAV	32 (38.1)	61 (37.0)	0.86	24 (40.7)	20 (33.9)	0.45
Organ damage, n (%)						
FFS = 0	2 (2.4)	10 (6.06)	0.2	2 (3.4)	2 (3.4)	1
FFS = 1	13 (15.5)	44 (26.7)	0.047	12 (20.3)	14 (23.7)	0.66
FFS ≧2	69 (82.1)	111 (67.3)	0.013	45 (76.3)	43 (72.9)	0.67
Maintenance dialysis	22 (26.2)	33 (20.0)	0.27	15 (25.4)	12 (20.3)	0.51
Interstitial pneumonia	14 (16.7)	48 (29.1)	0.032	12 (20.3)	11 (18.6)	0.82
Coexisting disease, no. (%)						
DM	24 (28.6)	50 (30.3)	0.78	19 (32.2)	15 (25.4)	0.42
Neurological disorder	6 (7.1)	12 (7.3)	0.97	2 (3.4)	3 (5.1)	0.65
Malignancy	4 (4.8)	18 (10.9)	0.11	3 (5.1)	3 (5.1)	1
Chronic pulmonary disease	5 (6.0)	15 (9.09)	0.39	5 (8.5)	7 (11.9)	0.54
Intervention, n (%)						
Mechanical ventilation	52 (61.9)	55 (33.3)	<0.001	31 (52.5)	33 (55.9)	0.71
Blood transfusion	45 (53.6)	54 (32.7)	0.002	26 (44.1)	28 (47.5)	0.71
ICU admission	35 (41.7)	27 (16.4)	<0.001	20 (33.9)	18 (30.5)	0.69
Remission induction treatment, n (%)						
Pulse steroid	82 (97.6)	133 (80.6)	0.0002	57 (96.6)	56 (94.9)	0.65
Cyclophosphamide	46 (54.8)	62 (37.6)	0.01	28 (47.5)	31 (52.5)	0.58
Rituximab	4 (4.8)	3 (1.8)	0.18	0 (0)	2 (3.4)	0.16

PE, plasma exchange; JCR, Japan College of Rheumatology; MPA, microscopic polyangiitis; GPA, granulomatosis with polyangiitis; AAV, antineutrophil cytoplasmic antibody-associated vasculitis; FFS, five-factor score; DM, diabetes mellitus; ICU, intensive care unit; IQR, interquartile range

The in-hospital mortality rate was significantly lower in the PE group compared to that in the non-PE group (35.6% vs. 54.2%, p = 0.041; risk difference, −18.6; 95% CI, −35.4% to −0.67%; Table 3). The number of patients with infection after hospitalization in the PE group was 20 (33.9%), and that in the non-PE group was 21 (35.6%) (p = 0.85; risk ratio, 0.95; 95% CI, 0.58–1.56), without a statistical difference (Table 4). The median length of hospitalization was 44 days (interquartile range, IQR, 27–84) in the PE group and 30 days (IQR, 16–58) in the non-PE group, reflecting a significantly longer duration in the PE group compared to that in the non-PE group (p = 0.027). Even when limited to the survivors, the median length of hospital stay was 49.5 days (IQR, 31–87.3) and 33 days (IQR, 23–62) for the PE and non-PE groups, respectively, with a significantly longer duration in the PE group compared to that in the non-PE group (p = 0.042).

**Table 3:** Comparison of mortality between the PE and non-PE groups.

	PE	Non-PE	P	Risk ratio (95% CI)	Risk difference (95% CI)
Matched cohort					
Overall in-hospital mortality	35.6% (21/59)	54.2% (32/59)	0.041	0.66 (0.43 to 0.99)	−0.19 (−0.35 to −0.01)
30-day in-hospital mortality	18.6% (11/59)	30.5% (18/59)	0.13	0.61 (0.32 to 1.18)	−0.12 (−0.27 to 0.04)
60-day in-hospital mortality	30.5% (18/59)	42.4% (25/59)	0.18	0.72 (0.44 to 1.17)	−0.12 (−0.28 to 0.06)
Pre-matched cohort					
Overall in-hospital mortality	41.7% (35/84)	34.6% (57/165)	0.27	1.21 (0.87 to 1.68)	0.07 (−0.06 to 0.20)
30-day in-hospital mortality	22.6% (19/84)	18.8% (31/165)	0.48	1.20 (0.73 to 2.00)	0.04 (−0.07 to 0.15)
60-day in-hospital mortality	36.9% (31/84)	27.3% (45/165)	0.12	1.35 (0.93 to 1.97)	0.10 (−0.03 to 0.22)

P values were estimated using the chi-square test. PE, plasma exchange; CI, confidence interval

**Table 4:** Incidence of infection after hospitalization in the PE and non-PE groups.

	PE	Non-PE	P	Risk ratio (95% CI)	Risk difference (95% CI)
Matched cohort					
Incidence of infection after hospitalization	20/59 (33.9)	21/59 (35.6)	0.85	0.95 (0.58 to 1.57)	−0.017 (−0.19 to 0.15)
Pre-matched cohort					
Incidence of infection after hospitalization	25/84 (29.8)	49/165 (29.7)	0.99	1.00 (0.67 to 1.50)	0.00065 (−0.12 to 0.12)

P values were estimated using the chi-square test. PE, plasma exchange; CI, confidence interval

### Factors associated with overall in-hospital mortality

There was no statistically significant difference in the in-hospital mortality between the PE and non-PE groups; however, PE therapy tended to decrease the risk of in-hospital mortality (odds ratio, OR, 0.41; 95% CI, 0.17–1.00; p = 0.05; Table 5). Univariate analyses revealed that age (65–75 years, >75 years), FFS ≥2, and the use of a mechanical ventilator were significant risk factors of in-hospital mortality. A multivariate analysis revealed that the use of a mechanical ventilator and age (>75 years) were independent risk factors of in-hospital mortality.

**Table 5:** Logistic regression analyses for in-hospital mortality in the propensity-matched patients.

	Univariate			Multivariate		
	OR	95% CI	P	OR	95% CI	p
Intervention						
Mechanical ventilation	7.46	3.22–17.28	<0.001	6.66	2.64–18.25	<0.001
ICU admission	2.17	0.99–4.77	0.051	1.28	1.28–0.46	0.63
Organ damage						
FFS						
FFS = 0 or 1	Reference					
FFS ≧2	2.88	1.16–7.16	0.02	1.3	0.34–4.83	0.69
Interstitial pneumonia	2.23	0.88–5.67	0.087	2.23	0.76–6.89	0.15
Age (years)						
<65	Reference					
65–75	4.5	1.31–15.46	0.013	3.92	0.85–22.09	0.081
>75	8.12	2.44–26.98	0.0002	5.73	1.27–30.70	0.023
Treatment						
PE	0.47	0.22–0.98	0.041	0.41	0.17–1.00	0.05

OR, odds ratio; CI, confidence interval; ICU, intensive care unit; FFS, five-factor score; PE, plasma exchange

## Discussion

The present study results suggest that PE therapy decreases the all-cause in-hospital mortality in patients with PH secondary to AAV. However, no difference was found between the PE and non-PE groups regarding the incidence of in-hospital infections. Furthermore, among all patients, including the survivors, the length of the hospital stay was longer in the PE group compared to that in the non-PE group. In addition, the risk factors of in-hospital mortality were advanced age (75 years and older) and the use of a mechanical ventilator. Although the use of PE therapy did not reach statistical significance in the multivariate logistic regression analysis for in-hospital mortality among propensity-matched patients, this factor tended to improve life expectancy.

PE therapy removes anti-neutrophil cytoplasmic antibodies involved in the pathogenesis of AAV from the plasma. Moreover, as it removes other proinflammatory factors, namely, complement factors, cytokines, and adhesion molecules (among others), it is a biologically reasonable treatment [27, 28]. The guidelines on AAV management prepared by the European League Against Rheumatism, European Renal Association, European Dialysis and Transplant Association, British Society for Rheumatology, and British Health Professionals in Rheumatology recommend PE therapy for severe renal impairment due to rapidly progressive glomerulonephritis and severe PH [4, 29]. The results of the present study suggest that 33.7% of patients with PH secondary to AAV receive PE therapy in Japan.

Although a previous case series demonstrated the efficacy of PE therapy in PH secondary to AAV in a monotherapy group of 20 patients [5], at present, no prospective comparative study has established an improvement in the acute phase prognosis by PE therapy in patients with PH secondary to AAV. In addition, among the several retrospective studies that have compared PE and non-PE groups, none conclude that PE therapy improves the acute phase prognosis in patients with PH secondary to AAV [6–10]. Thus, presently, the evidence on the efficacy of PE therapy on improving the acute phase prognosis in patients with PH secondary to AAV is poor.

However, the present study results suggest that PE therapy decreases the all-cause in-hospital mortality in patients with PH secondary to AAV. Although statistical significance was not reached, PE therapy tended to decrease the in-hospital mortality risk. The results of the present study may differ from those of previous studies for the following two reasons. The first reason concerns the number of patients and their backgrounds. The present study included a large number of patients from many institutions (194 hospitals), reflecting the present medical environment. In addition, it was restricted to patients requiring proactive therapy. In contrast, the preceding studies involved either a single institution or a small number of specialized institutions, and the study cohort comprised a small number of patients. Furthermore, even in PH secondary to AAV, there are reports of patients with mild symptoms that have no correlation to the risk of mortality [30, 31]. Some of the preceding studies included patients without decreased oxygenation or who were not on oxygen treatment, as well as those receiving outpatient treatment. In a recent study involving patients in a single institution using a strict diagnosis of PH secondary to AAV, the lowest mortality rate for the incidence of death-related events was 11% [6], which is exceedingly low compared to that of the present study (44.9%) and those of the preceding studies (17.0%–58.3%) [7–10].

The second reason the present results may differ from those in previous studies concerns the baseline adjustment of the PE and non-PE groups. Generally, the likelihood that severely ill patients will receive PE therapy is high. Therefore, confounding by indication might have affected the results in previous studies. Even in the present study, patients who underwent PE therapy mostly tended to be severely ill patients who were predicted to have a very poor prognosis (e.g. FFS ≥2, using a mechanical ventilator, undergoing blood transfusions, and admitted to the ICU) and those receiving proactive therapy (steroid-pulse therapy and cyclophosphamide therapy). By using a propensity score matching analysis, we were able to create PE and non-PE groups that were balanced on important background characteristics. Specifically, the factors considered as likely to have affected the treatment choice, which were adjusted to obtain a balance between the groups in the present study, were (1) AAV-induced organ disorder; (2) patient management for more severe cases; (3) factors found to be correlated with mortality in previous studies (age and FFS) [13]; (4) available medical facilities, as suggested from the characteristics of this study and routine medical care; and (5) the main comorbidities that affect treatment choice but are not related to AAV. In most of the previous studies, a baseline adjustment was not applied between the PE and non-PE groups. However, in a recent single-institution study with the highest number of patients to date, propensity score matching was used in the baseline adjustment of both groups [6]. This previous study included the Birmingham Vasculitis Activity Score for Wegener's Granulomatosis and C-reactive protein levels [6], which were not included in the present study; rather, we included respiratory failure, renal failure, and the use of renal replacement therapy, cyclophosphamide treatment, and rituximab treatment. Furthermore, adjustments were made in the present study using more detailed items that likely affect the treatment choice, obtaining a better balance between the groups.

The PE and non-PE groups did not differ in the incidence of in-hospital infection. As PE therapy removes immunoproteins, such as immunoglobulins, complement proteins, and cytokines, it theoretically increases susceptibility to infections. However, even in previous studies, an increase in the rate of infections has not been observed.

Among all patients, excluding those who expired in the hospital, the length of stay was significantly longer in patients who underwent PE therapy compared to that in the patients who did not undergo PE therapy. Thus, we could not demonstrate that PE therapy shortens the duration of the in-hospital stay. No previous study has investigated this point. The reason for the prolonged in-hospital time is unknown; further research may clarify the issue.

In patients with PH secondary to AAV, the risk factors for in-hospital mortality were age (75 years or older) and the use of mechanical ventilation. The results of the multivariate logistic regression analysis for in-hospital mortality in propensity-matched patients suggest that PE therapy is likely a factor that reduces the mortality rate. Being elderly and using mechanical ventilation were also found to be long-term mortality risk factors in patients with PH secondary to AAV patients in a previous study [13]. Patients with a high FFS and those undergoing dialysis were shown to have a higher risk of death in this previous study; however, we did not reach such a conclusion, as the present study assessed the acute phase prognosis over a short observation period. Moreover, severely ill patients with an FFS ≥2 accounted for 72.3% of the patients in the present study, comprising the majority of the eligible patients, which may have influenced the results.

### Limitations

The present study has some limitations. First, this was a retrospective observational study and, therefore, it was not randomized. Although the patients’ baseline characteristics were adjusted via propensity score matching, the results may be biased because of confounding factors that still cannot be measured. Second, the accuracy of the AAV diagnosis is a limitation. As the present study was a retrospective investigation based on information obtained from an administrative database, we could not confirm whether each patient met the classification criteria. However, the diagnosis of AAV for many patients was made and validated by a specialist in Japan, as these patients receive assistance with medical expenses from the government through a review based on fixed standards [32]; thus, the data can be considered reliable to some extent. Third, vital signs, laboratory data details, and the patients’ physical condition could not be confirmed from the DPC data. Therefore, the Birmingham Vasculitis Activity Score and accurate diagnosis of PH could not be confirmed. However, in the present study, AAV and the underlying PH diseases were confirmed by the attending physicians. Patients with diseases that would need to be distinguished from PH secondary to AAV were excluded.

## Conclusions

The present study demonstrates that PE therapy improves all-cause in-hospital mortality in patients with PH secondary to AAV. As recommended in the existing guidelines, PE therapy can be considered for patients with severe PH secondary to AAV.
